# While flattening the curve and raising the line, Africa should not forget street vending practices

**DOI:** 10.34172/hpp.2021.05

**Published:** 2021-02-07

**Authors:** Isaac Olushola Ogunkola, Uchenna Frank Imo, Hope Jonah Obia, Elvis Anyaehiechukwu Okolie, Don Eliseo Lucero-Prisno III

**Affiliations:** ^1^Department of Public Health, University of Calabar, Calabar, Nigeria; ^2^Department of Public Health, Teesside University, Middlesbrough, United Kingdom; ^3^Department of Global Health and Development, London School of Hygiene and Tropical Medicine, London, United Kingdom

**Keywords:** Africa, COVID-19, Street vendor

## Abstract

Street vending practices are common in Africa and cater to a large portion of the continent’s population. Since the identification of coronavirus disease 2019 (COVID-19) in Africa, various governments have implemented measures to control the spread of infection. These measures may have an adverse effect on street vending practices. This paper demonstrates the extent to which COVID-19 measures of control and prevention affects street vending practices in Africa and how it can be remedied. There has been reduced economic growth and increased hunger among individuals involved in street vending practices due to the prohibitions put in place by governments. Measures directed at curbing the spread of the virus inadvertently affect street vending practices and vendors. Current and future pandemic response plans should reflect the integration of measures directed at reducing potential hardship and a further economic set back for individuals involved in street vending practices.

## Introduction

Practices that provide needed goods and services for the population which are controlled and practised by the informal sector most time, exist as street vending practices.^1^ The informal sector accounts for over 80% of service provision in a typical African economy, particularly in the provision of basic needs such as food.^2^ With the advent of the coronavirus disease 2019 (COVID-19) pandemic across the globe, there have been measures adopted by the government and health systems in curbing the spread of the virus. With a large proportion of Africa’s business persons into or related to different street vending practices, the effect of such adopted measures in curbing the spread of COVID-19 pandemic has either suspended or reduced street vending activities. This poses a threat to the wellbeing and potential survival of street vendors. This is because the suspension of street vending practices precipitates a sharp decline in the income of street vendors. This paper seeks to highlight the economic impact of COVID-19 response measures in Africa on street vendors. Furthermore, it will recommend approaches that may be crucial in addressing potential disadvantages faced by street vendors as a result of varying COVID-19 response measures in the continent.

## Efforts to flatten

Although Africa as a continent has been battling with different epidemics, the emergence of COVID-19 in the continent provides a different perspective to the already existing battles in the continent.^3,4^ In retrospect, Africa’s response to the Ebola epidemic in 2018 received rave reviews. It has been suggested that such response positions the continent to identify gaps in preventing, detecting, and responding to similar public health threats in the future.^3^ Globally, 42 879 817 cases have been confirmed with over 1 million deaths across different regions as at 30th of November. There are also reports of decreased incidence and mortality cases since the third quarter of 2020. The increased level of misinformation in circulation before the emergence of the pandemic in the continent has prolonged the containment phase of the disease in many African countries.^3^ Over the months of the raging pandemic, health systems in Africa have responded to the pandemic using different measures focused on the reduction of spread through testing, contact tracing, isolation, and treatment of infected persons. The continent had also looked towards the possibility of producing a vaccine for the infection. Some of the major measures put in place include full or partial lockdown especially in urban centres, suspension of street vending practices, and closure of places that could be overcrowded such as religious centres, schools, and markets, and the improvement of health services.^1^ Importantly, the establishment of genome sequencing laboratories reflects significant strides among health systems in Africa in efforts to contain the COVID-19 pandemic.^1,5^

## Means of economic survival

Currently, massive urbanization reflected in rural-urban migration is pervasive in Africa. Such skewed flow of migration to the urban areas is fuelled by the search for a better quality of life.^6^ Unfortunately, academic qualification is a vital factor needed to secure a moderate paying job in the urban region and this has left many who migrated without these qualifications to resort to the very low paying jobs such as cleaners, gatekeepers, drivers etc. A study that was carried out in Ikorodu Car Park, Lagos State, Nigeria, revealed that majority of the vendors are 21-30 years of age, consisting mostly of males with secondary school education as their highest academic qualification.^7^ Alternatively, those who find these jobs demeaning decide to go into street vending as an alternative means to earn an income especially in populated cities with high service provision needs. A broad range of goods and services are provided by street vendors ranging from food, clothes, shoes and home appliances etc. They can be seen along the streets, in traffic, along walkways & footpaths, local markets or even carts delivered at home.^6^

Overtime street vending has become very lucrative as people who live in urban centres are almost always on the go and spend most of their time in traffic where these vendors are concentrated. In Kenya, street vending is categorized under Small and Micro Enterprises which provides Jobs to over 70% of the Kenyan population, street vending contributes a lot to the economy but is not accounted for because it is not seen as part of the nation’s economic sources.^8^ Street vending practices have been proven to cater for the needs of the vendors and also provide resources especially food for a greater population in Africa.

## Impact of COVID-19 on street vending practices

There is a strong relationship between street vending practices and COVID-19 transmission.^9^ The nature of the association between vendors and most consumers who patronise their businesses increases the chances of the spread. In a bid to reduce the spread of the virus, African governments have implemented various measures which may be unfavourable for these vendors but beneficial to their health. Social distancing, self-isolation and the travel bans as measures of curbing the spread of COVID-19 have led to a drastic reduction in the activities of the vendors. Additionally, most governments have also closed down the local markets and streets where these vendors are found to mitigate the rate of spread.^1^ These measures affect the economic status and wellbeing of these vendors. Most effects could be long term. In restricting the activities of these vendors, there would be unavoidable economic setbacks which may usher poverty and severe lack. There may be increased health risks and criminal offences when vendors are laid out of their activities of survival. Children and other vulnerable groups could suffer from these limitations experienced by the vendors.

Street vending contributes substantially to the African economy. However, its continued practice would invariably increase the susceptibility of the vendors to the coronavirus. Previous studies have demonstrated that the pattern of enforcement of health-related restrictions during disease outbreaks in Africa portends unfavourable outcomes for members of the informal sector including street vendors.^10^ Lockdowns and other social distancing measures cause major problems for both consumers and vendors. However in some countries like Uganda, for instance, food vendors were allowed to stay in markets if they were willing to sleepover rather than travel back home each evening.^1,11^ But to increase social distancing, non-food vendors were removed. As a result of the enforcement of such lockdown restrictions by the government, the vulnerability of Uganda’s street vendors increased.^9^ Similarly, it has been reported that millions of Nigerians observing the lockdown lack both food and income that will allow the survival of their families.^2^ In Ghana, it has been reported that the income of market traders including the street vendors has dropped by 90% due to the pandemic. The report also has it that 90% of Zimbabwean survive on the informal economy such as street vending.

In Africa, informal traders often have a frail social contract with the government. Such frail contract aggravated their vulnerability during the pandemic. The COVID-19 pandemic has left policymakers with a focal challenge to choose between uncontrolled outbreaks of the virus or to implement social distancing with a concomitant negative impact on the livelihood of street vendors.

## A way forward

With the efforts of the African governments to reduce the rate of spread of the virus and increase the number of people who recover from the infection, there is a need to address sectors like street vending practices. A fundamental mandate of the government is the protection of people’s rights and livelihood. In line with social security principles, African governments should ensure certain provisions are made to palliate present difficulties faced by street vendors. These provisions may include relief materials, food, and conditional cash transfer.^2^ Additionally, the formation of a collaborative partnership between stakeholders in street vending practices and relevant authorities provides an opportunity for consolidating recorded achievements in the pandemic response. This collaborative partnership will allow for the development of sustainable plans that are favourable for street vending practices, ensures the continuous supply of goods and services to consumers, and are compliant to established prevention protocols against COVID-19.^1,10^ Also, a motorised awareness campaign should be used to reach out to street vendors commonly found on major roads and highways.

Community-based strategies should be adopted to increase the responsiveness of vendors and the possibility to thrive amidst major setback. Community health education and promotion programmes should be organised to train vendors on the best obtainable practices to avoid further spread of the virus during their activities. Typical discussions should be conducted among groups of vendors to understand how best to address the problems in this new normal and evaluate the implemented solutions. Vendors should be encouraged to set up bodies that will collate funds to provide for response materials such as sanitizers, masks etc. Vendors should also be taught how to make most response materials like sanitizers, nose masks, face shield etc. in their immediate environment. This will help save the cost of external acquisition and will also drive sustainable solutions from the vendors in their environment. Peer to peer support should be encouraged and rewarded by vendor committees in the market to effect behavioural change and increase the uptake of COVID-19 precautionary measures.

There are possibilities of further limitations that may be faced by these vendors. Health ministry and primary care agencies in the neighbourhood should partner with unions of vendors to measure the level of adoption of the recommended practice. This will serve to address issues of misconception among vendors. It will also help vendors abide by the best preventive practices. Loan investments should be set up by the government and capable financial institutions to support businesses of vendors who are affected by the pandemic. Cuts and discounts should be made on the levies and taxes paid by the vendors. Such subsidy will serve as a support in extenuating the current difficulties plaguing their businesses. To placate the economic effects of COVID-19, governments should set up schemes that will encourage the populace to buy from street vendors. Innovative techniques like mobile kiosk should be recommended to safeguard jobs and upscale sales as vendors observe the safety guidelines for COVID-19 prevention.

Furthermore, day(s) for sanitation should be allotted in a week to ensure a high level of hygiene in places of food vending practices,^10^ and protective measures among street vendors should be introduced.^12^Government should liaise with local tailors to provide free nose masks to both buyers and sellers in markets There should be strategic placement of hand-washing apparatus, distribution of PPEs, use of sanitizers and nose masks, proper spacing and distancing among vendors etc. The need for these mechanisms should be emphasized as the only means of not depriving street vendors their source of livelihood. This will bring about adherence and improved advocacy for these measures. Importantly, there is a need to strengthen data collection in informal sectors across the continent including street vending practices.^6^ This will ensure that future disease containment plans and policies of government acknowledge the importance of street vendors and other informal sectors in the implementation of certain measures complexities inherent in epidemic response efforts point to the need for plans to be flexible to accommodate unpredicted issues.

## Conclusion

In responding to COVID-19 in Africa, certain harms may be done to groups that solely survive from informal sectors of the economy. Interestingly, these groups constitute a large proportion of the population in the continent. Particularly, street vending practices serve as a source of income and livelihood for many Africans. As such, where measures implemented by the government is antagonistic to street vending practices, street vendors may experience economic hardship. The extent to which the economy of most African nations are supported by street vending practices including food vending highlights the need for government to make decisions that positively impact the sector and individuals involved. Failure in adhering to these considerations may result in further hardship and an economic setback for the continent.

## Acknowledgements

The authors appreciate the reviewers for their insightful comments.

## Funding

No funding received.

## Competing interests

The authors declare that they have no competing interests.

## Ethical approval

Not applicable.

## Authors’ contributions

IOO, UFI, HJO developed the concept and prepared the initial draft. EAO and DEL assisted with data collection and edited the draft. All authors approved the final version.
